# Diamond Jubilee of Stoke Park Research Centre

**Published:** 1990-09

**Authors:** J. Jancar

**Affiliations:** Honorary Consultant Psychiatrist, Stoke Park Hospital, Stapleton, Bristol BS16 1QU


### Diamond Jubilee of Stoke Park Research Centre

**J. Jancar. MB BCh BAO FRCPsych DPM**
Honorary Consultant Psychiatrist, Stoke Park Hospital, Stapleton, Bristol BS16 1QU

*"Stoke Park with associated Hospitals have a long record of illustrious research into the field of Mental Handicap, associated with such famous names as Berry, Norman, Fraser Roberts, Golla and Grey Walter, and also an outstanding record for the provision of services for the mentally handicapped . . . I consider that the field of mental handicap is one of the most promising areas of psychiatry for research, and we can look forward to most exciting and important developments in this field."*

Professor Linford Rees—President of the Royal College of Psychiatrists—at the official opening of the Polyclinic at Purdown Hospital on 20th May, 1977.

The founder of Stoke Park Colony, the Reverend Harold N Burden was not content merely to offer custodial care for mentally handicapped patients but made financial provision for and encouraged research into causes, treatment, prevention and care of mental handicap.

In 1930 Mr. Burden appointd Professor R. J. A. Berry as Director of Medical Services at Stoke Park—the first research centre—and as Chairman of the Burden Research Trust. Just before his death on 15th May, 1930, Mr. Burden approved a scheme for further development of research work at Stoke Park and increased the financial provision far beyond the more modest scale first suggested.

Professor Berry was a man of high distinction who created for himself a reputation in two hemispheres and also in the fields of medical science. He had already made a name at Edinburgh among British anatomists when, at the age of 38, he went to occupy the Chair of Anatomy in the University of Melbourne. In Australia he became interested in the problem of mental handicap, and eventually became honorary consultant psychiatrist to the Melbourne Hospital and honorary psychiatrist to the Children's Hospital in that city. He also contributed to the literature on the subject, including Stewart lectures on medical psychology, delivered in Melbourne, and Beattie-Smith lectures on insanity in Melbourne and at Cornell University, New York. Professor Berry also published a report on mental handicap in the State of Victoria.

Professor Berry's approach to mental handicap was global and multi-professional. His approach continues to this day in Stoke park.

Throughout 60 years the staff, who were all full-time apppointees, were totally committed to this specialty in respect of patients of all ages and disabilities. They were clinically orientated under the medical leadership but free to research in their own disciplines. They exchanged their findings and new ideas between disciplines but never slavishlly followed unproven philosophy or change for the sake of change.

The Research Centre gradually became the "Stoke Park School of Mental Handicap", where undergraduate and postgraduate students and visitors from this country and abroad were able to learn about the latest scientific research and approaches to mental handicap.

However, the care of the patients and their families was always of paramount importance. The provision of community care for mentally handicapped and the avoidance of hospital admission whenever possible, was always the aim at Stoke Park—long before community care became "fashionable". Since 1958 out-patient clinics in local Health Authorities and general hospitals were established. In 1961 an Assessment Unit at Hanham Hall was opened and in 1977, to improve out-patient, in-patient and research facilities, the new Polyclinic was built for Frenchay Health District at Purdown Hospital.

In the eighties, total rebuilding of Stoke Park, as an integral part of the community, was completed and now offers modern facilities for in-patients, temporary respite care, and out-patients, suitable for mentally handicapped people of all ages and mental and physical disabilities who require hospital care within the Frenchay Health Authority catchment area.

Stoke Park was one of the first centres to recognise the importance of research and provision of services for ageing mentally handicapped people which is now becoming an increasingly serious problem. In 1987 a National Symposium on ageing was organised in Bristol.

Stoke Park Hospital staff were always actively involved in the Royal Medico-Psychological Association and some were senior officers of RMPA. Members of the hospital staff strongly supported the creation of the Royal College of Psychiatrists and two are founder fellows of the College. Since the establishment of the College in 1971 the following offices were held by the staff: Two vice-presidents, one Chairman of South Western Division, three Chairman of Section of Psychiatry of Mental Handicap, and one Chairman of the Mental Handicap Specialist Advisory Committee of the J.C.H.P.T. Two members were Blake-Marsh lecturers and one was elected as an honorary Fellow of the College. This great recognition and distinction of Stoke Park and its staff will not be easily surpassed.

The future of research and undergrauate and postgraduate education at Stoke Park by medical and non-medical staff is assured with the joint establishment of an academic department of mental handicap in 1982 between the University of Bristol Mental Health Department and Stoke Park Hospital.

Professionalism and enthusiasm of the present staff will no doubt carry the ideals of Stoke Park pioneers into the 21st century and beyond.

The span of 60 years in time means very little but 60 years of research at Stoke park are packed with pioneering work in all spheres of mental handicap as evident from over 460 publications (1) which will no doubt leave a mark in the history of mental handicap in this country.

It is interesting to note that the first paper from the Research Centre, by Berry (2), was published in the Bristol Medico-Chiruriginal Journal in 1930. Its title, "Hospitals—Voluntary or Self-Supporting?", could be appropriate for the 1990s—Nothing new under the sun!

### REFERENCES

1. JANCAR, J. (1990). Stoke Park Publications (1930–1990) Mental Handicap. (In Press).
2. BERRY, R. J. A. (1930). Hospitals—Voluntary or Self-Supporting? *Bristol Medico-Chirurgical Journal*, **47**, 19.

